# GRN, NOTCH3, FN1, and PINK1 expression in eutopic endometrium – potential biomarkers in the detection of endometriosis – a pilot study

**DOI:** 10.1007/s10815-020-01905-4

**Published:** 2020-10-07

**Authors:** Isabell Holzer, Amanda Machado Weber, Anne Marshall, Alexander Freis, Julia Jauckus, Thomas Strowitzki, Ariane Germeyer

**Affiliations:** Department of Gynaecological Endocrinology and Fertility Disorders, University Hospital Heidelberg, Heidelberg, Germany

**Keywords:** Endometriosis, Biomarkers, Progranulin, Fibronectin

## Abstract

**Purpose:**

Endometriosis (EM) is a common gynecological disease affecting 10–15% of women of reproductive age. However, molecular mechanisms and pathogenesis are still not completely understood. Furthermore, due to the absence of a reliable clinical biomarker, the only viable method for the often-delayed definitive diagnosis is laparoscopic surgery. Our objective was to analyze molecular differences of selected endometrial proteins and genes of women suffering from different stages of EM compared with healthy women to evaluate potential clinical biomarkers.

**Methods:**

We analyzed eutopic endometrial tissue samples from women undergoing a laparoscopic surgery (*n* = 58). mRNA gene expression of progranulin (*GRN*), neurogenic locus notch homolog protein (*NOTCH3*), fibronectin (*FN1*), and PTEN-induced kinase 1 (*PINK1*) was analyzed using qRT-PCR. Protein expression was determined using ELISA and immunohistochemistry.

**Results:**

Significant differences in gene expression between the different stages of the disease were noted for GRN, NOTCH3, FN1, and PINK1 (*p* < 0.05). The endometrium of women with minimal EM (ASRM I) showed the highest mRNA expression. Protein levels of GRN and FN1 on the other hand were significantly decreased in the endometrium of women with EM compared with those of healthy controls. Furthermore, for GRN and FN1, we could detect a correlation of protein expression with the severity of the disease.

**Conclusion:**

Our findings suggest a potential use of GRN and FN1 as clinical biomarkers to detect endometriosis. In addition, GRN, NOTCH3, FN1, and PINK1 could potentially be useful to differentiate between the underlying stages of the disease. However, a validation with a larger study population is needed.

## Introduction

Endometriosis (EM) is defined as the presence of functional endometrium in any location outside the uterine cavity. The ectopic endometrial cells can be present in the ovaries, peritoneum, and fallopian tubes and even in distant organs such as the lung and brain [15]. EM typically causes dysmenorrhea, dyspareunia, and pelvic pain and may contribute to infertility [9]. It affects approximately 10–15% of women of reproductive age and can even be found in 35–50% of women suffering from pelvic pain and/or infertility [14]. However, the prevalence might be underestimated due to non-specific symptomatology and late diagnosis caused by a lack of non-invasive methods for detecting. According to recent estimates, the diagnosis of EM is delayed by an average of 7–11 years [18]. So far, there is no reliable clinical marker and the gold standard diagnostic method is invasive laparoscopy [19]. EM can be graded according to the revised American Society for Reproductive Medicine (rASRM) staging system in minimal, mild, moderate, and severe stages of the disease (ASRM I–IV), depending on the character, expansion, and localization of the lesions as well as the presence of adhesions [16]. It is the most common international classification of EM; however, the infiltration depth and severity of symptoms are not included in this classification. None of the patients included as ASRM I and II had a deep infiltration.

Although various theories about the pathogenesis of EM have been submitted, none of them is proven so far and the exact causes and pathogenetic pathways of this heterogenetic disease are still not fully understood [42]. Furthermore, it has been suggested that within the disease, there are distinct entities and pathogenesis [24]. Clinical studies have shown different implantation rates in women with mild versus severe EM, assuming a difference in function and biochemistry of these two stages of EM [20].

Although EM is considered a benign disease, it shares several characteristics with malignancy including excessive proliferation, cellular invasion, peripheral metastasis, inflammation, and estrogen dependency [21]. Patients with EM even suffer a slightly increased risk of developing ovarian carcinomas, especially of the endometrioid and clear cell subtypes [1, 7, 28].

Considering these circumstances, EM is one of the most common female health disorders and has a great impact on the patient’s quality of life [36]. Thus, it is necessary to identify potential molecular biomarkers for diagnosis and regulatory factors, underlying the progress of EM.

Herein, the objective of our study was to investigate molecular differences in the expression of specific genes and proteins in eutopic endometrium from women with and without EM. We analyzed changes of these proteins and genes in different stages of the disease in an attempt to determine the degree of the disease without the necessity of an abdominal surgery. We wanted to validate the assumption of a potential instinct pathogenesis and entity of mild and severe endometriosis by showing a difference in gene and protein expression. Furthermore, we tried to investigate proteins that have the potential for use as clinical biomarkers to enable an earlier diagnosis without the need for laparoscopy. We focused on proteins that are already known to be involved in inflammatory diseases, cell adhesion, and migration as they are assumed to play an important role in the pathogenesis of endometriosis. Furthermore, we wanted to investigate proteins that have already been described in connection with implantation, cell development, or blastocyst maturation. Therefore, we chose to examine the gene and protein expression of progranulin (GRN), neurogenic locus notch homolog protein (NOTCH3), fibronectin (FN1), and phosphatase and tensin homolog–induced kinase 1 (PINK1).

## Materials and methods

### Sample collection and patients

Endometrial biopsies were taken from women (32.8 ± 4.23 years old) undergoing a laparoscopic surgery. The procedure was performed for benign reason due to unexplained pelvic pain or sterility. Exclusion criteria were hormonal stimulation within the last 3 months, endocrinopathies, cancerous lesions, and irregular menstrual bleeding. All biopsies were taken in the mid-to-late proliferative phase and collected in TRIzol for mRNA analysis (*n* = 40), in frozen nitrogen for protein analysis (*n* = 40), and in O.C.T.™ for immunohistostaining (*n* = 18). Endometriosis was graded according to the revised American Society for Reproductive Medicine (rASRM) staging system in minimal (< 5 points; ASRM I), mild (6–15 points, ASRM II), and moderate EM (16–40 points, ASRM III). None of the patients included as ASRM I and II had deep infiltrating disease*.* As a control, we used samples of women also suffering from pelvic pain or sterility but for reasons other than EM. The groups did not differ significantly in size, nor in age, nor from the day of the menstrual cycle. All participants gave their informed consent for the use of samples under the approved ethics protocol of the Ruprecht Karls University Heidelberg (S239/2005).

### mRNA analysis via qRT-PCR

The RNA was isolated using TRIzol® reagent (Ambion® by Life Technologies, USA) according to the manufacturer’s instructions. The concentration and purity of the mRNA were detected using a NanoDrop spectrometer (NanoDrop, ND-1000, USA). A total of 1 μg of total RNA was reverse-transcribed to synthesize the complementary DNA (cDNA) using the AMV Reverse Transcriptase (Promega, Germany). The mRNA expression was performed using TaqMan primers of *GRN*, *NOTCH3*, *FN1*, and *PINK1* and TaqMan universal PCR master mix (Applied Biosystems, Germany). For analysis, the real-time quantitative reverse transcription polymerase chain reaction (qRT-PCR) on a 7500 Fast Real-Time PCR System (Applied Biosystems, UK) was used. Amplification was initiated with 10-min incubation at 95 °C followed by 40 cycles of 15 s at 95 °C and 1 min at 60 °C. The results were calculated using the ΔΔ*C*_T_ method and expressed as a fold-change between the control (non-endometriosis endometrium) and the different EM groups. *RPL0* was used as a housekeeping gene for the normalization of the *C*_T_ values. The following primers were used: *GRN* (Hs00173570_m1, Thermo Fisher Scientific, Germany), *NOTCH3* (Hs01128541_m1, Thermo Fisher Scientific, Germany), *FN1* (Hs001549976_m1, Thermo Fisher Scientific, Germany), *PINK1* (Hs00260868_m1, Thermo Fisher Scientific, Germany), *RPL0* (Hs99999902_m1, Thermo Fisher Scientific, Germany).

### Immunohistochemistry

For immunostaining, the tissues frozen in O.C.T.™ Compound (Tissue Tek, Netherlands) were sliced in 7-μm serial sections and fixed with acetone at 4 °C. After rinsing the sections in PBS (Gibco, USA) (2 × 5 min), the unspecific bindings were blocked using a blocking solution (Candor Bioscience, Germany). The sections were incubated overnight at 4 °C with an unconjugated specific primary antibody. The endogenous peroxidase was blocked with 0.3% H_2_O_2_ in TBS (1 l deionized water, 6.05 g Tris, 8.76 g NaCl, pH 7.5). The sections were incubated with a biotinylated secondary antibody for 30 min to 1 h depending on the antibody and followed by a 30-min incubation with a streptavidin-peroxidase complex (Vectastain, UK). After rinsing in PBS (2 × 2 min), staining was visualized with an AEC Plus substrate solution (Dako, USA) for 15 min followed by a counterstaining with hematoxylin (Dako, Germany). Following another rinse with water, the sections were dehydrated at room temperature and mounted with Aquatex (Merck, Germany). Negative controls were performed omitting the primary antibody. The following primary and secondary antibodies in mentioned concentrations were used: anti-PGRN (goat IgG, 1:200, AF2420, R&D Systems, USA, RRID:AB_2114489), anti-NOTCH3 (rabbit IgG, 1:200, ab23426, Abcam, USA, RRID:AB_776841), anti-FN1 (mouse IgG, 1:200, ab6328, Abcam, USA, RRID:AB_305428), anti-PINK1 (mouse IgG, 1:50, ab186303, Abcam, USA, RRID:AB_2827698), anti-goat IgG (1:200, PK-1605, Vectastain, UK), anti-rabbit IgG (1:500, TD268284, Dako, USA), anti-mouse IgG (1:300, PK-6102, Vectastain, UK).

The samples were evaluated by two independent observers who were blinded to the sample background. The expression of the proteins was classified as the product of defined percentage and the intensity called intensity–reactivity score (IRS). The percentage of staining cells were specified as follows: 0 for no documented positive staining cell; 1 for < 10% positive staining cells; 2 for 10–50%; 3 for 50–70%, and 4 for > 80%. Moreover, in terms of intensity of the stain, the following scores were designated: 0 for no marked stains; 1 for weak; 2 for moderate, and 3 for high intensity of staining.

### Protein analysis via ELISA

Protein extracts were obtained from samples by tissue homogenization in lysis buffer (Cloud-Clone Corp., USA) and centrifugation for 10 min at 2590×/*g* at 4 °C followed by 5 min at 5000×/*g* at 4 °C according to the manufacturer’s instructions. Protein concentration was determined by Bradford protein assay kit (Bio-Rad, Germany). Enzyme-linked immunosorbent assay (ELISA) was performed to quantify the protein levels of GRN, NOTCH3, FN1, and PINK1. Standards, samples, reagents, and microplate preparations were performed as described by the manufacturer. Optical density of each well was measured using a microplate reader at a wavelength of 450 nm (Anthos Labtec Instruments, Austria). All conditions were measured in duplicates. Protein concentrations were calculated using the mean of the duplicates in relation to the total protein amount. The following kits were used: Progranulin ELISA Kit (No. E-EL-H1578, Elabscience, USA), Human Notch Homolog 3 ELISA Kit (No. SEL147Hu, Cloud-Clone Corp., USA), Quantikine ELISA Human Fibronectin (No. DFBN10, R&D Systems, USA), Human PINK1 ELISA Kit (No. MBS9327222, MyBioSource, USA).

### Statistical analysis

SPSS® version 24.0 (IBM SPPS, USA) was used to perform statistical analyses. The continuous variables were expressed as mean ± standard deviations (SEM). Descriptive analysis presents the median, range, and standard deviation (SD). Unpaired Student’s *t* test was conducted for numerical variable analysis. Normal distribution was tested by the *Kolmogorov*–*Smirnov test.* For non-parametric analysis, we applied the Mann–Whitney *U* test. *p* < 0.05 was defined as statistically significant.

## Results

### Patients’ characteristics

In total, endometrial samples of 58 women were divided according to the degree of underlying EM into four categories: women without EM were used as control; women with minimal (ASRM I), mild (ASRM II), and moderate (ASRM III) EM. As shown in Table 1, there was no significant difference noted regarding female age, parity, gravity, number of previous abortions, and day of menstrual cycle when the biopsy was taken between the groups.

**Table 1:** Age and cycle day profile with standard deviation of the subgroups

	All	Control	ASRM I	ASRM II	ASRM III
*n*	58	18	16	11	13
Mean age	32.77	32.94	32.47	32.91	32.77
± SEM	0.56	1.15	1.25	0.74	1.09
Minimum age	21	21	23	30	25
Maximum age	41	41	41	37	37
Mean cycle day	10.83	11.88	10.80	10.55	10.08
± SEM	0.32	0.80	0.46	0.68	0.42

### GRN, NOTCH, FN1, and PINK1 gene expression

**Fig. 1:**
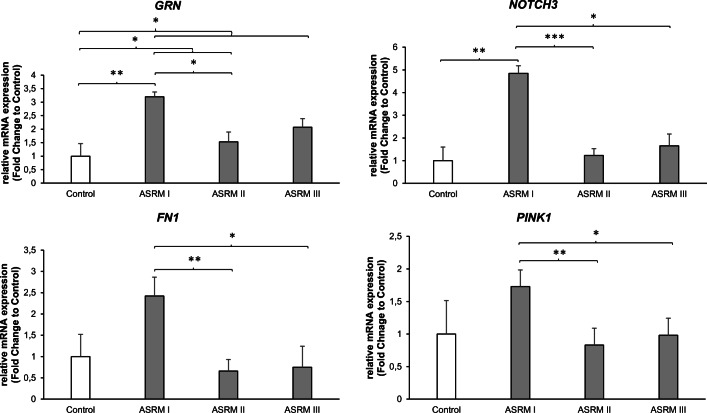
Changes in mRNA expression levels. Relative mRNA expression levels of *GRN, NOTCH3, FN1,* and *PINK1 *to the house keeping gene *RPL0*. Bar graphs showing gene expression levels relative to the control group. Data represents mean fold change ± SEM. Control (without EM, *n* = 10); ASRM I (minimal EM, *n* = 10); ASRM II (mild EM, *n* = 10); ASRM III (moderate EM, *n* = 10). *p < 0.05; **p < 0.01; ***p < 0.001

To analyze the molecular differences in gene expression among the groups, we assessed the mRNA expression levels of *GRN*, *NOTCH3*, *FN1*, and *PINK1*. Descriptive data of gene expression is presented in panel 1 of Table 2. Figure 1 shows the significant effect of changes in gene expression according to the severity of the disease. In all assessed protein coding genes, mRNA expression in women with minimal EM (ASRM I) seems to be overexpressed compared with the others.

**Table 2:** Dataset of conducted experiments with some descriptive statistics showing the sample size, median, range*,* and standard deviation

	Control			ASRM I			ASRM II			ASRM III		
	Median	Range	SD	Median	Range	SD	Median	Range	SD	Median	Range	SD
Panel 1: PCR* (*n* = 40)												
GRN	12.08	3.65	1.48	10.12	1.61	0.56	11.24	3.60	1.15	10.60	3.09	1.02
NOTCH 3	10.72	5.40	1.89	8.02	2.98	1.04	9.98	2.73	0.94	9.14	5.22	1.66
FN 1	10.07	4.74	1.65	8.87	4.67	1.41	10.37	2.80	0.87	10.15	4.89	1.55
PINK 1	10.01	5.23	1.62	9.31	2.66	0.81	10.43	3.03	0.82	10.06	2.22	0.84
Panel 2: IHC* (*n* = 18)												
GRN	3.50	2.13	0.86	3.38	2.38	0.94	3.75	3.13	1.71	3.88	3.13	1.22
NOTCH 3	4.17	5.67	2.49	4.67	3.83	1.43	7.33	1.83	0.93	5.83	6.67	2.55
FN 1	11.00	3.00	1.34	9.00	4.00	1.64	11.00	1.00	0.58	12.00	2.00	1.10
PINK 1	2.5	5.00	2.12	3.00	4.75	2.02	5.00	2.00	1.00	2.05	4.25	1.97
Panel 3: ELISA* (*n* = 40)												
GRN	110.57	182.69	60.00	60.38	192.96	54.02	70.19	137.02	39.66	60.09	88.99	26.92
FN1	108.08	53.44	17.49	102.33	125.76	35.89	80.79	86.19	26.22	75.20	63.34	19.05

*Unit: the results of PCR are shown in Δ*C*_т_; the intensity–reactivity score was used to measure IHC; the results of ELISA are presented in pg/mg for GRN and ng/mg for FN1

The gene expression of *GRN* within eutopic endometrium is significantly increased in the minimal EM disease (ASRM I) compared with the control (*p* < 0.01). Furthermore, a reduction in expression in the higher stages of the disease compared with minimal disease is seen, reaching a significant downregulation in ASRM II compared with ASRM I (*p* < 0.05). Last, but not the least, comparing all assessed EM groups with the control group, a significant higher gene expression of *GRN* was noted in women with EM (*p* < 0.05).

*NOTCH3* demonstrated a similar gene expression pattern within eutopic endometrium with a significant increase in expression in minimal disease (ASRM I) compared with the healthy control (*p* < 0.01). In addition, *NOTCH3* is differentially expressed within the different degrees of the disease with a significant decrease (nearly to the extent of the control) in ASRM II (*p* < 0.001) and ASRM III (*p* < 0.05) compared with ASRM I.

While the gene expression of FN1 and PINK1 in minimal disease (ASRM I) was increased compared with that in the healthy control, as mentioned prior, this did not reach significance. But again, we found a significant decrease in gene expression of both genes between ASRM II compared with ASRM I (*p* < 0.01) as well as ASRM III compared with ASRM I (*p* < 0.05).

### Immunoreactivity of GRN, NOTCH3, FN1, and PINK1

**Fig. 2:**
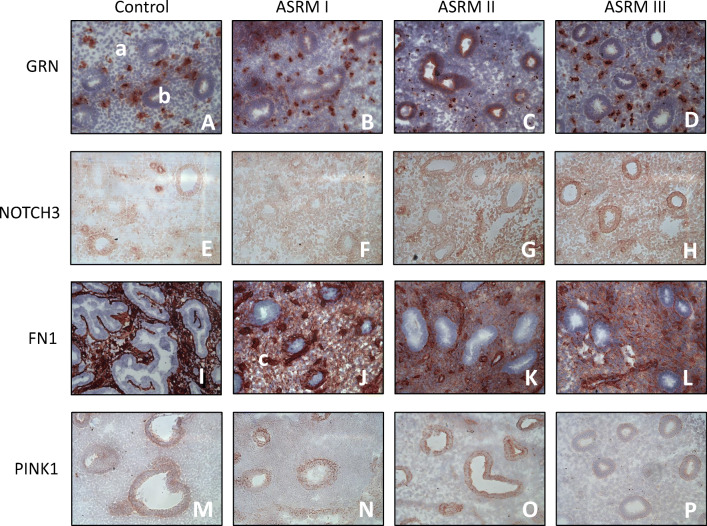
Immunohistochemical staining in tissue samples of different EM stages. Representative images (× 20) of GRN (A–D), NOTCH3 (E–H), FN1 (I–L), and PINK1 (M–P) protein expression by immunohistochemistry in endometrium of women without (control—A, E, I, M) (*n* = 5), minimal (ASRM I—B, F, J, N) (*n* = 5), mild (ASRM II—C, G, K, O) (*n* = 3), and moderate EM (ASRM III—D, H, L, P) (*n* = 5). The images demonstrate the typical staining differences in stromal (a), epithelium (b), and endothelium (c) cells of eutopic endometrium tissue depending on the severity of EM

Immunohistochemistry demonstrated a significant level of all examined proteins in every eutopic endometrial tissue sample. Figure 2 depicts some exemplary cases of the immunohistochemical staining for GRN, NOTCH3, FN1, and PINK1 in endometrial tissue without EM as well as minimal, mild, and moderate EM. The descriptive data of the IHC is shown in panel 2 of Table 2.

GRN seemed to be strongly expressed in the cytoplasm of stromal cells compared with that of epithelial cells; nevertheless, a difference in expression could only be noted in the compartment of the epithelial cells between the groups of EM. Particularly epithelial cells of women with ASRM II seemed to be stained more than in women with other stages of the disease or the control.

NOTCH3 was expressed in the nuclear compartment but not in the cytoplasm of the cells. While the expression in stromal cells was slightly noted, NOTCH3 seemed to be more dominantly expressed in epithelial and endothelial cells. Although the staining of NOTCH3 did not change significantly between the different groups, it seemed to be more pronounced in women with ASRM II.

**Fig. 3:**
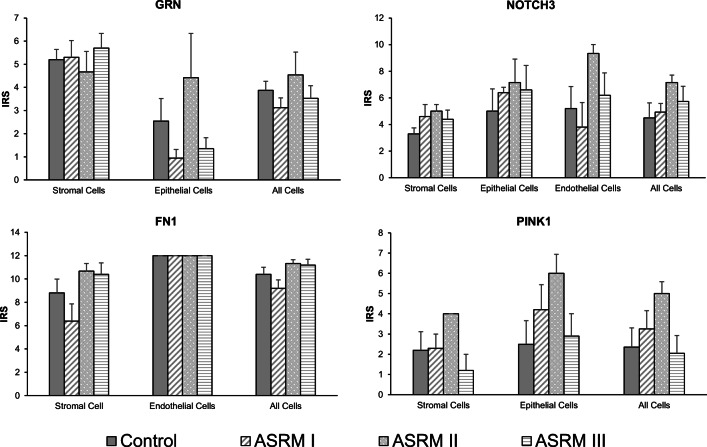
Immunoreactivity and localization of proteins in eutopic endometrial tissue samples. Immunohistochemistry for GRN, NOTCH3, FN1, and PINK1. Data presents the mean of the intensity–reactivity score (IRS) of each cell type stained with the assessed proteins compared by the stage of EM. Bar graphs showing the typical localization of the determined proteins in eutopic endometrial tissue. IRS, intensity–reactivity score (product of intensity of staining and percentage of stained cells); intensity of staining: 0, none; 1, mild; 2, moderate; 3, high; percentage of stained cells: 0, none; 1, < 10%; 2, 10–50%; 3, 50–80%; 4 > 80%. Data represents the mean ± SEM. Control (without EM, *n* = 5); ASRM I (minimal EM, *n* = 5); ASRM II (mild EM, *n* = 3); ASRM III (moderate EM, *n* = 5)

The expression of FN1 was dominantly noted in stromal and endothelial cells in every endometrial tissue but not in epithelial cells. As shown in Fig. 3, no significant differences were seen regarding the intensity of staining of FN1 in endothelial cells between the different groups. However, a tendency is observed on stronger expression in stromal cells of women with mild and moderate EM.

The staining of PINK1 was noted in both cytoplasm and nucleoplasm and seemed to be greatly expressed in epithelial than in stromal cells. Although there was no significance found among the different groups, we could detect the most dominant expression in endometrial tissue of women with ASRM II and the lowest in ASRM III samples

### Lower GRN and FN1 protein expression in patients with EM

**Fig. 4:**
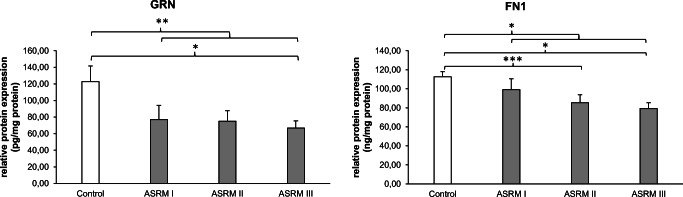
Changes in protein expression levels. Results of enzyme-linked immunosorbent assay (ELISA) of human eutopic endometrial tissue. Relative protein expression levels of GRN and FN1 to the total protein amount of the endometrial tissue sample. Bar graphs showing protein amount in pg (GRN) or ng (FN1) per 1 mg of total protein amount. Data represents the mean ± SEM. Control (without EM, *n* = 10); ASRM I (minimal EM, *n* = 10); ASRM II (mild EM, *n* = 10); ASRM III (moderate EM, *n* = 10). *p < 0.05; **p < 0.01; ***p < 0.001

We performed enzyme-linked immunosorbent assay (ELISA) to obtain a quantitative analysis about the differences in protein expression between the four assessed groups: no EM, ASRM I, ASRM II, and ASRM III. Descriptive data for protein expression is presented in panel 3 of Table 2. Figure 4 shows the significant effects of changes in protein levels of GRN and FN1 for each stage of the disease. While the protein levels of NOTCH 3 were undetectable, PINK1 had very low quantities of secreted protein and was only detectable in 12 out of 40 samples. This is in agreement with our IHC findings, where NOTCH3 and PINK1 showed only slight expression especially in the cytoplasm.

Sufficient protein expression however was found in GRN, which demonstrated a significantly lower expression in women with EM compared with women without EM (*p* < 0.01). Moreover, the protein expression decreased depending on the severity of the disease. The higher the stage of EM, the lower the concentration of GRN in eutopic endometrial tissue. In endometrial tissues of healthy women (122.74 ± 18.97 pg/mg), GRN was on average 38% higher expressed than in women with ASRM I (76.92 ± 17.08 pg/mg). The expression was even lower in women with ASRM II (75.08 ± 12.54) and reduced 45% in women with ASRM III (66.83 ± 8.51 pg/mg, *p* < 0.05). However, values for GRN expression were not normally distributed. In order to test the robustness of our results, we conducted additional non-parametric tests (Mann–Whitney *U* test). All results remain to be significant.^1^

The same pattern was seen in the relative protein expression of FN1. Our data shows that the protein expression of FN1 is significantly downregulated in women with EM in comparison with that in women without EM (*p* < 0.05). The mean concentration of FN1 protein amount in the whole endometrium sample was reduced 12% among women with minimal EM (99.19 ± 11.35 ng/mg), 25% for women with mild EM (85.36 ± 8.29 ng/mg; *p* < 0.001), and even 30% among women with moderate EM (79.28 ± 6.02 ng/mg; *p* < 0.05) compared with women without EM (112.55 ± 5.53 ng/mg).

## Discussion

In this study, we show differences in protein and gene expression of selected genes among the different degrees of EM, according to the ASRM classification, as well as compared with the endometrium of healthy controls. For the first time, we demonstrate an association of GRN, NOTCH3, FN1, and PINK1 with the disease of EM. Their role in other tissues and physiological or pathological mechanisms has been described in many other studies [3, 10, 30, 39]; the regulation of the selected proteins in human EM however remains to be examined.

GRN is a pleiotropic glycoprotein and growth factor with proliferative, invasive, and anti-inflammatory properties [17]. Despite the fact that a couple of studies have attributed GRN a role within blastocyst development and implantation of mice and mink [12, 13, 31], very little is known of its function in human endometrium. In our study, GRN mRNA expression is considerably upregulated in women with EM compared with that in healthy controls, which may contribute to its role in inflammation and cell proliferation process. Moreover, our findings of GRN protein expression show a significant continuous reduction in expression level the higher the degree of the disease. While *Qin et al.* demonstrated that the addition of recombinant GRN in blastocyst culture media promoted blastocyst hatching, adhesion, and outgrowth, rabbit anti-mouse GRN IgG reduced that effect [31]. The reduction of GRN in the endometrium of women with EM may have a negative effect on blastocyst development, potentially contributing to a reduction of implantation rates in affected women, as seen in women with EM according to the severity of the disease [37].

There are few studies revealing Notch expression in the endometrium [2, 25, 26]. It is known that the Notch signaling pathway plays a fundamental role in the development of diverse organisms including mammals [3]. Currently, the Notch system is believed to impact differentiation, proliferation, and apoptosis and last but not the least influence organ formation and morphogenesis [3]. In addition, the Notch genes seem to influence the regulation and remodeling of the vascular system [34], including angiogenesis in endometrial cancer [27]. As little is known about the function of Notch signaling in the reproductive tract, this is the first study showing the effects of NOTCH3 in EM. Similar to the findings of *Mikhailik et al.* (25) describing NOTCH 1–3 expression in both, endometrial stromal and epithelial cells, we also observed a positive staining in these two cell types. Furthermore, while *Shawber et al.* described NOTCH3 as the only Notch ligand not expressed in capillary endothelial cells but in pericytes in the preimplantation uterus of mice [35], we found a moderate immunopositivity of NOTCH3 in the endothelium of endometrial blood vessels. Similar to the findings of *Cobellis et al.* showing a nuclear expression of NOTCH1 and NOTCH4 in the endometrium [11], we also detected positive immunostaining of NOTCH3 exclusively in the nuclear cell compartment. Therefore, our results indicate that not only NOTCH1 and 4 but also NOTCH3 plays an important role in angiogenesis of the endometrium. Our investigations on mRNA level exhibited a particular elevated level of *NOTCH3* gene expression in women with minimal EM compared with women without EM, as well as an overall elevated expression. The fact that Notch signaling protein expression is increased in human endometrial carcinoma cells compared with that in healthy endometrial cells [27] is in line with the assumed effect of NOTCH3 in proliferation and differentiation [3]. Furthermore, this observation poses again the question of the parallel operations between EM and malignancy. Caused by its overexpression in several types of cancers, NOTCH3 has already been investigated as a target for anticancer drugs [6]. In addition, recent studies showed an activation of the Notch domain and its abnormal signaling in connection with chronic inflammatory diseases leading to pathological fibrotic processes [22], which underlines the possible relevance of NOTCH3 in the development of EM. Therefore, our findings of overexpression of NOTCH3 in patients with EM may open a new perspective of NOTCH3 as a target for EM therapy, particularly in early disease.

FN1 is a glycoprotein of the extracellular matrix, which is involved in important cellular mechanisms including cell adhesion, migration, wound healing, blood coagulation, and even metastasis [30]. Some studies already analyzed FN1 and its role in the endometrium and implantation [8, 40, 41], as well as in pathogenesis of EM [29, 32]. *Sapkota et al.* identified in a meta-analysis of SNPs the association of the FN1 locus with EM, mainly with moderate-to-severe EM [32]. Based on these genomic findings, we wanted to investigate the role of FN1 directly in eutopic endometrial tissue of women with EM. Others, like *Beliard et al.*, suggested FN1 could have a function in persistence of endometriotic lesions and found elevated FN1 receptor expression in the endometriotic glands in peritoneal lesion compared with the eutopic endometrium of healthy controls [5]. While they had a small number of eutopic and ectopic tissues during different phases of the menstrual cycle, they did not find a difference in FN1 expression between the tissues [5]. In contrast to their study, we used only mid-to-late proliferative samples with clearly determined stages of EM disease, where we were able to detect changes in protein expression showing a stage-dependent reduction of FN1 expression, suggesting that not only the receptors are regulated in endometrium of women with EM, but also the FN1 protein itself plays a role in endometriosis. Furthermore, while *Beliard* *et al.* demonstrated an upregulation of FN receptor within epithelium of ectopic lesions [5], FN1 as seen in our IHC is exclusively expressed in the capillary endothelium and endometrial stromal cells and not in the epithelium. The localization of FN1 in the endothelium could potentially contribute to its role in angiogenesis that is depicted in tumor pathogenesis [23]. As FN1 and its receptors are believed to be important in mammalian reproduction and placentation [8], with a particular impact on blastocyst adhesion and implantation [40], the dysregulation of FN1 in the eutopic endometrium of women with EM may potentially affect the implantation potential of patients with EM. However, this remains to be further analyzed.

Mutations in PINK1 seem to play an important role in the pathogenesis of Parkinson’s disease [38]. Besides that, little is known about PINK1’s function in other human tissues. Herein, we investigated for the first time the role of PINK1 in the eutopic human endometrium of women with and without EM. Our data show a mild expression of PINK1 in stromal cells and a moderate expression in the glandular epithelium of the human endometrium of healthy women as well as in the endometrium of all different types of EM. We even could find a mild increase, however not significant, in women with endometriosis ASRM I and even further with ASRM II, which gets lost in ASRM III. Interesting enough, *Barodia et al*. describe that a deficit of PINK1 results in increased mitochondrial autophagy leading to an accumulation of reactive oxygen radicals [4]. Recent studies defined oxidative stress as an important factor in the pathophysiology of endometriosis due to an imbalance between reactive oxygen species and antioxidants, resulting in an inflammatory response within the peritoneal cavity [33]. While we do not observe a reduction in PINK1 expression in women with EM, we experience a dysregulation of PINK1 with a particular high gene expression in minimal disease that vanishes over the progression of the disease. The importance of this finding within the proliferative phase of the cycle remains to be elucidated further.

To the best of our knowledge, this is the first study assessing GRN, NOTCH3, FN1, and PINK1 among the different degrees of EM. We could show differential expression of these genes and proteins within the different stages of EM as well as to healthy endometrium. These findings may support the suggestion of a potentially distinct entity and physiopathology between minimal, mild, and moderate EM already notable within the eutopic endometrium. Furthermore, our results open a new field of interest regarding potential biomarkers of EM as well as a better understanding of differences in gene and protein expression in women with EM. However, a prospective validation is required for the actual function of the investigated proteins and genes in EM and their use as clinical biomarkers.

## Footnotes

1 Results of the Mann–Whitney *U* test (exemplary only significant values are shown): GRN—control versus ASRM II: *p* = 0.043, control versus ASRM III: *p* = 0.011, control versus EM: *p* = 0.006; FN1—control versus ASRM II: *p* = 0.009, control versus ASRM III: *p* = 0.003, control versus EM: *p* = 0,006.

## Abbreviations

ELISA: enzyme-linked immunosorbent assay
EM: endometriosis
FN1: fibronectin 1
GRN: progranulin
NOTCH3: neurogenic locus notch homolog protein 3
PINK1: phosphatase and tensin homolog–induced kinase 1
PBS: phosphate-buffered saline
qRT-PCR: real-time quantitative reverse transcription polymerase chain reaction
TBS: Tris-buffered saline
